# Comparative study of total thoracoscopic and traditional incision internal fixation for the treatment of multiple rib fractures

**DOI:** 10.1007/s00068-025-02994-5

**Published:** 2025-11-14

**Authors:** Kun Yan, Liang Chu, Zhe Zhang, Song Wu, Guiping Yu, Zhonghua Qin

**Affiliations:** 1 Department of Cardiothoracic Surgery, Jiangyin Clinical College of Xuzhou Medical University, Wuxi Jiangsu, 214400 China; 2 Department of Thoracic and Cardiac Surgery, the First Clinical College of Xuzhou Medical University, Xuzhou Jiangsu, 221000 China

**Keywords:** Total thoracoscopic, Traditional incision, Internal fixation, Rib fracture, Surgery

## Abstract

**Objective:**

To investigate the clinical efficacy of total thoracoscopic memory alloy embracing fixator(MAEF) internal fixation surgery for multiple rib fractures.

**Methods:**

A retrospective analysis was conducted on the clinical data of 83 patients with multiple rib fractures who underwent surgical treatment in our department from September 2020 to February 2025. The patients were divided into an observation group (total thoracoscopic group, *n* = 40) and a control group (traditional incision group, *n* = 43) according to the surgical method. The clinical data of the two groups were compared.

**Results:**

The operative time in the observation group was longer. However, after stratification according to the learning curve, it was found that the operative time in the observation group showed no significant difference compared with the control group after the surgical technique became proficient (*P* > 0.05). The pain index and partial pressure of oxygen one day before surgery, intraoperative blood loss, postoperative pleural drainage volume, and fracture healing status in the observation group showed no significant differences compared with the control group (*P* > 0.05). Pain scores and oxygen partial pressures on postoperative days 3 and 5 were significantly better in the observation group than in the control group(*P* < 0.05). The postoperative hospital stay duration was significantly shorter in the observation group than in the control group (*P* < 0.05).

**Conclusion:**

The total thoracoscopic MAEF internal fixation surgery for multiple rib fractures is easy to learn, causes minimal trauma, and allows for a rapid postoperative recovery. The fracture healing achieved is comparable to that of open thoracotomy surgery.

## Background

Multiple rib fractures are a common type of chest trauma, often leading to chest wall instability and paradoxical respiratory movement, which severely affect the patient’s respiratory function and quality of life [1, 2]. The traditional surgical treatment method is internal fixation through a small incision, which can effectively stabilize the fractured ends and restore chest wall stability. However, this approach is associated with significant surgical trauma, marked postoperative pain, longer recovery time, and a higher incidence of complications. With the development of minimally invasive techniques, thoracoscopic internal fixation for rib fractures has been increasingly reported, but most of these reports focus on fixation of the external surface of the ribs [3–5]. Zhang Jijun reported a new surgical method of total thoracoscopic internal fixation of rib fractures, which provides a new treatment strategy for patients with rib fractures [6]. Currently, there are relatively few studies on this surgical approach [7–9]. This study aimed to compare the clinical outcomes of total thoracoscopic and traditional incision internal fixation for multiple rib fractures by retrospectively analyzing the clinical data of patients treated with these two methods, in order to provide evidence for the selection of clinical treatment plans.

## Materials and methods

### General information

In 2023, our department began to perform total thoracoscopic internal fixation surgery for rib fractures. Prior to this, patients with rib fractures were treated with traditional incision internal fixation surgery. A retrospective analysis was conducted on the data of 83 patients with rib fractures who underwent surgical treatment in our hospital’s thoracic surgery department from September 2020 to February 2025. All patients enrolled in this study presented with multiple rib fractures, defined as fractures in three or more ribs. Surgical intervention was indicated if any of the following conditions were present: [1] flail chest or thoracic wall collapse; [2] concurrent pulmonary contusion, hemothorax, or atelectasis with hypoxemia; [3] markedly displaced rib fractures accompanied by severe pain. This study was approved by the Ethics Committee Board of Jiangyin People’s Hospital.

Inclusion criteria: [1] Patients met the diagnostic criteria for multiple rib fractures and were confirmed by imaging examinations such as X-ray and CT;[2] Chest AIS score ≥ 3 and underwent surgical treatment; [3] Age between 18 and 70 years; [4] No severe cardiopulmonary dysfunction; [5] No coagulation dysfunction; [6] No contraindications to surgery; [7] Complete clinical data.

Exclusion criteria: [1] Accompanied by other severe thoracic injuries requiring emergency surgery (e.g., cardiac or major vascular trauma, progressive hemothorax, etc.); [2] History of previous thoracic surgery; [3] Presence of severe extra-thoracic organ injuries; [4] Patients who refused to participate in this study.

Based on the surgical method, the patients were divided into an observation group (total thoracoscopic internal fixation surgery) and a control group (traditional incision internal fixation surgery), with 40 cases in the observation group and 43 cases in the control group. In the observation group, 4 patients had flail chest and 13 sustained additional injuries: finger fractures, toe fractures, cerebral concussion, orbital fracture, and lumbar vertebral fracture. In the control group, 2 patients had flail chest and 17 had accompanying injuries: finger fractures, toe fractures, nasal-bone fracture, ankle fracture, mild thoracic-compression fracture, and cerebral contusion. There was no significant difference in the general information between the two groups (*P* > 0.05), and the groups were comparable (Table 1). This study has been approved by the Medical Ethics Committee of Jiangyin People’s Hospital.

**Table 1:** Comparison of general information between the two groups of cases

	Observation Group(*n* = 40)	Control Group(*n* = 43)	t	Ρ
Gender, Male	30(75%)	31(72.1)		0.808
Age(years)	52.55 ± 10.25	53.14 ± 12.44	−0.235	0.815
Fracture ends (sites)	5.13 ± 1.34	4.63 ± 1.36	1.672	0.098
Flail chest(n,%)	4(10%)	2(4.7%)		0.422
Associated injuries(n,%)	13(32.5%)	17(39.5%)		0.648
Fracture site(left/right)	22/18	27/16		0.509
ISS score	6.13 ± 4.54	6.72 ± 4.76	−0.582	0.562

### Surgical methods

After completing all investigations and a comprehensive evaluation, a surgical plan was formulated for every patient; operations were performed within 5 days of admission. All procedures were carried out under general anaesthesia with a double-lumen endotracheal tube.

**Fig. 1:**
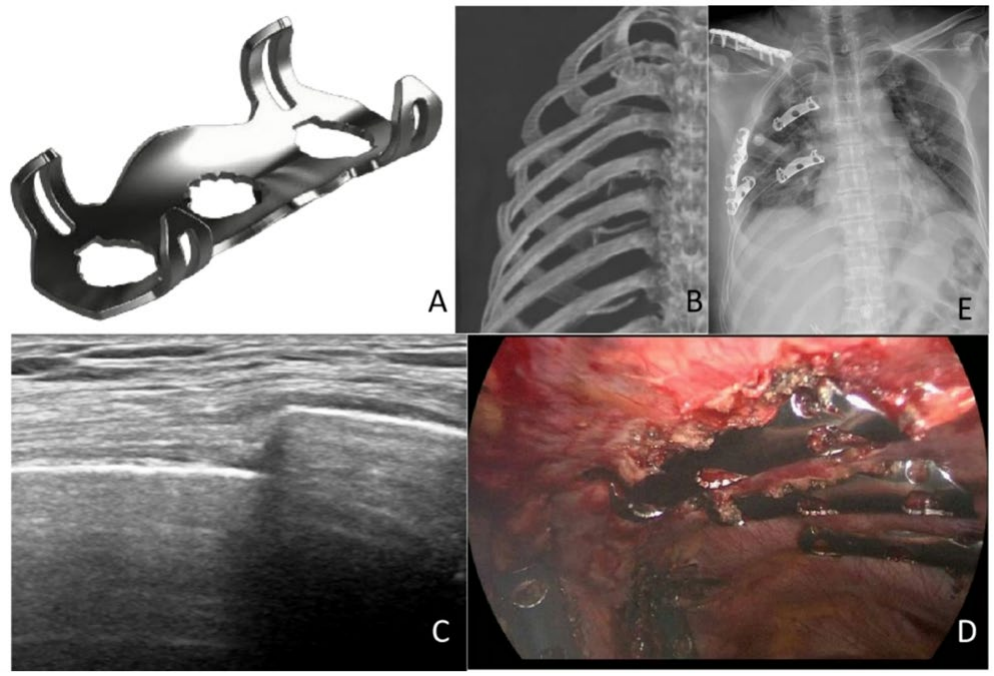
**A** A reverse Memory Alloy Embracing Fixator(Ximai International Medical Co., Ltd., Lanzhou, China); **B** Preoperative 3D reconstruction image of right-sided multiple rib fractures; **C** Preoperative high-frequency ultrasound localization image of rib fractures; **D** Postoperative image of right-sided multiple rib fractures fixed with reverse memory alloy embracing fixators; **E** Chest X-ray following internal fixation of right-sided multiple rib fractures

In the observation group, the rib fracture ends were fixed using a reverse Memory Alloy Embracing Fixator (MAEF) under total thoracoscopy (Ximai International Medical Co., Ltd., Lanzhou, China)(Fig. 1A). Depending on the fracture location, 1 to 3 minimally invasive small incisions were selected. After positioning the patient for surgery, high-frequency ultrasound was used to locate the rib fracture ends and mark them on the body surface(Fig. 1B-C). If the rib fracture ends were covered by the scapula, the fracture ends were located based on preoperative CT and anatomical structures. The approximate fracture site is first identified with high-frequency ultrasound landmarks or anatomical reference. The parietal pleura was incised along the longitudinal mid-axis of the fractured rib to expose the cortical bone; after identifying the fracture ends, dissection was extended superiorly and inferiorly to reveal the upper and lower margins of the rib, and an additional 3 cm of exposure was obtained on both the proximal and distal sides of the fracture. A custom-made long clamp with a heart-shaped tip was used as a lever to reduce the displaced fracture ends by focal, controlled pressure. An appropriately sized reverse MAEF was then selected. The fixing claws of the MAEF were expanded in disinfected ice saline at 0–4℃ and quickly placed on the rib surface to cover the fracture ends. Subsequently, warm saline was injected onto the surface of the MAEF to contract the embracing fixator, ensuring a reliable fixation (Fig. 1D-E).

**Fig. 2:**
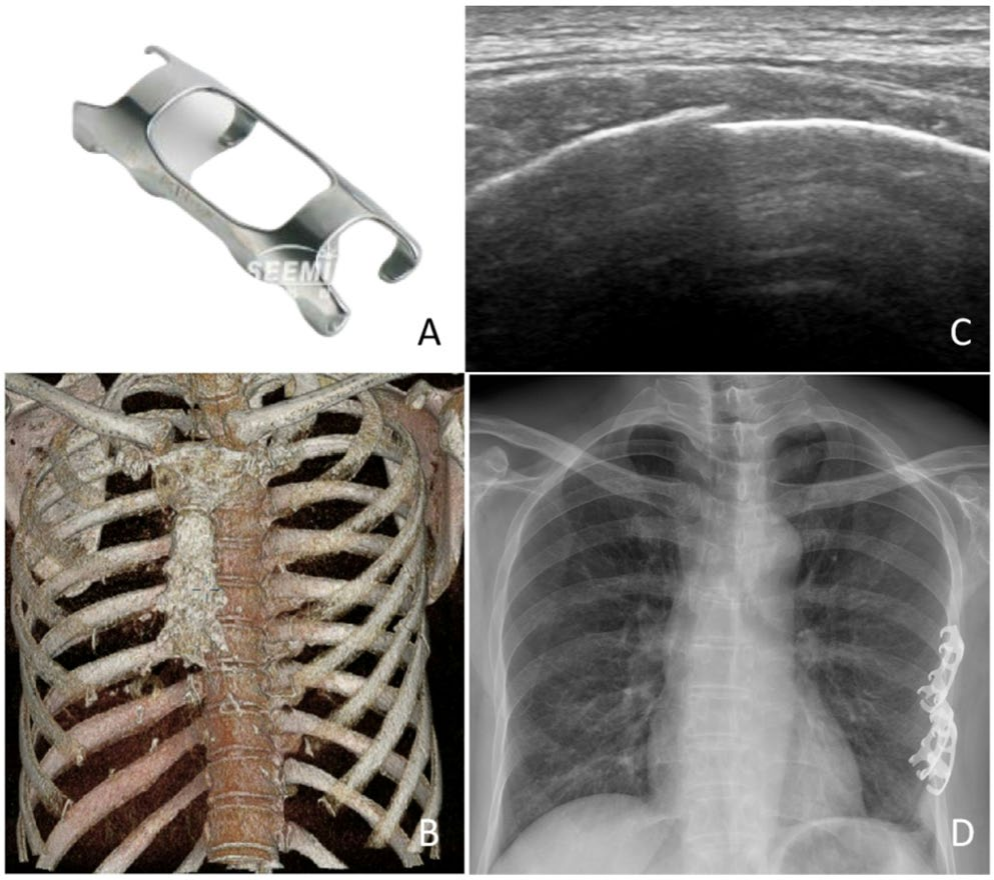
**A** A Memory Alloy Embracing Fixator(Ximai International Medical Co., Ltd., Lanzhou, China); **B** Preoperative 3D reconstruction image of left-sided multiple rib fractures; **C** Preoperative high-frequency ultrasound localization image of rib fractures; **D** Chest X-ray following internal fixation of left-sided multiple rib fractures

In the control group, the rib fracture ends were fixed using MAEF (Ximai International Medical Co., Ltd., Lanzhou, China)(Fig. 2A) through conventional open surgery. After positioning the patient for surgery, high-frequency ultrasound was used to locate the rib fracture ends and mark them on the body surface(Fig. 2B-C). If the rib fracture ends were covered by the scapula, the fracture ends were located based on preoperative CT and anatomical structures. The surgical incision was selected according to the fracture location, and the incision had to fully expose the fracture ends. After the fracture was reduced, an appropriately sized MAEF was selected. The fixing claws of the MAEF were expanded in disinfected ice saline at 0–4℃ and quickly placed on the fracture site. After confirming the correct position of the embracing fixator, warm saline was applied to contract the fixator, which tightly embraced the fracture ends (Fig. 2D).

### Observation indicators

Surgical-related indicators: The preoperative blood oxygen indicators (1 day before surgery, blood samples were collected for analysis 20 min after discontinuation of oxygen therapy), operative time, intraoperative blood loss, postoperative pleural drainage volume, and postoperative hospital stay duration were recorded for both groups of patients.

Pain intensity: The pain intensity of patients was assessed using the Visual Analogue Scale (VAS) 1 day before surgery, 3 days after surgery, and 5 days after surgery. The score ranges from 0 to 10, with higher scores indicating more severe pain. Throughout the peri-operative period every patient was managed with a multimodal analgesic strategy: [1] Intravenous NSAIDs: fast-onset, high-intensity relief for acute pain immediately after injury or in the early post-operative period; [2] Oral NSAIDs or weak opioids: maintenance analgesia during the stable phase after surgery; [3] Transdermal patches: low-dose, continuous release for long-term maintenance once the patient is stable.For operated patients, the anesthesiologist also provides a patient-controlled analgesia (PCA) pump loaded with an opioid for on-demand pain control.

Postoperative recovery status: The postoperative hospital stay duration, blood oxygen indicators on postoperative day 3 and day 5 (blood samples were collected for analysis 20 min after discontinuation of oxygen therapy), and fracture healing status at 3 months after surgery (assessed by X-ray or CT examination) were observed for both groups of patients. Fracture healing was evaluated as follows: [1] Good – fracture well united with satisfactory alignment and no thoracic deformity; [2] Fair – fracture united with mild displacement but no thoracic deformity; [3] Poor – any result not meeting the above criteria.

Complications: The incidence and recovery of postoperative complications (such as pulmonary infection, bleeding, pneumothorax, pleural effusion, and atelectasis) were documented for both groups of patients.

### Statistical analysis

Statistical analysis was performed using SPSS 25.0 software. Continuous data were expressed as mean ± standard deviation (‾x ± s), and intergroup comparisons were conducted using independent samples t-test. Categorical data were presented as percentages, and intergroup comparisons were performed using Fisher’s exact test. A P value less than 0.05 was considered to indicate a statistically significant difference.

## Results

All operations were completed without incident. Post-operative complications occurred in 8 patients (20%) in the observation group: two developed bacterial pneumonia because of ineffective expectoration, three had atelectasis due to pain-related reluctance to cough, one accumulated pleural fluid after chest-tube malfunction, and two experienced new-onset atrial fibrillation. In the control group, 5 patients (11.6%) experienced complications: one pneumonia from poor sputum clearance, two atelectasis for the same pain-related reason, one small pneumothorax after tube removal, and one episode of atrial fibrillation. The difference in complication rates between the two groups was not statistically significant (*P* > 0.05) (Table 2). All complications resolved with appropriate management, and every patient was discharged in stable condition.

**Table 2:** Comparison of postoperative data between the two groups of cases

	Observation Group(*n* = 40)	Control Group (*n* = 43)	t	Ρ
Operative time (minutes)	133.30 ± 24.68	109.02 ± 24.81	4.465	0.000
Incidence of complications (n,%)	8(20%)	5(11.6%)		0.371
Intraoperative blood loss (ml)	73.93 ± 13.52	72.56 ± 16.05	0.418	0.677
Preoperative pain index on Day 1	7.43 ± 1.04	7.49 ± 1.07	−0.273	0.786
Postoperative pain index on Day 3	6.03 ± 0.70	6.40 ± 0.82	−2.207	0.030
Postoperative pain index on Day 5	4.95 ± 0.93	5.42 ± 0.79	−2.471	0.016
Preoperative PO₂ on Day 1(mmHg)	77.83 ± 7.69	78.51 ± 6.15	−0.451	0.653
Postoperative PO₂ on Day 3(mmHg)	87.60 ± 6.23	83.14 ± 6.78	3.113	0.003
Postoperative PO₂ on Day 5(mmHg)	92.43 ± 7.49	88.93 ± 6.35	2.298	0.024
Postoperative hospital stay duration(days)	7.60 ± 1.46	8.30 ± 1.51	−2.153	0.034
Postoperative pleural drainage volume(ml)	280.88 ± 104.98	312.33 ± 101.23	−1.389	0.169
Fracture healing status (good/fair)	26/14	35/8		0.135

In the observation group, the first 20 cases were defined as the initial stage, and the last 20 cases were defined as the proficient stage according to the learning curve. Although the total operative time in the observation group was longer than that in the control group (*P* < 0.05), the operative time in the observation group was similar to that in the control group after proficiency was achieved, with no significant difference between the two groups (*P* > 0.05) (Table 3). There were no significant differences between the observation group and the control group in terms of intraoperative blood loss, postoperative pleural drainage volume, and fracture healing status (*P* > 0.05). The postoperative hospital stay duration was shorter in the observation group than in the control group, with a significant difference between the two groups (*P* < 0.05). There were no significant differences between the observation group and the control group in the pain index and partial pressure of oxygen 1 day before surgery (*P* > 0.05). However, the pain index on postoperative day 3 and day 5 was significantly lower in the observation group than in the control group, and the partial pressure of oxygen on postoperative day 3 and day 5 was higher in the observation group than in the control group, with significant differences between the two groups (*P* < 0.05) (Table 2).

**Table 3:** Comparison of operative time according to different learning curves

	Observation Group Initial Phase(*n* = 20)	Observation Group Proficient Phase(*n* = 20)	Observation Group Proficient Phase(*n* = 20)	Control Group (*n* = 43)
Operative time (minutes)	150.40 ± 18.96	116.20 ± 16.60	116.20 ± 16.60	109.02 ± 24.81
*t*	6.070		1.175	
*Ρ*	0.000		0.245	

## Discussion

The total thoracoscopic intrathoracic internal fixation surgery for rib fractures reported by Zhang Jijun is a completely new surgical procedure [6]. Since thoracoscopic techniques are already well-established for most thoracic surgeons, experienced thoracic surgical teams can easily adopt total thoracoscopic internal fixation surgery. The only aspect that requires adaptation is the proper use of the special instruments. The total thoracoscopic MAEF requires the use of specific instruments for placement and support. The surgeon and assistant need to work in close coordination to perfectly position the MAEF. If the angle of the rib fracture is particularly unusual, it will further increase the difficulty of properly placing the MAEF.

In this study, the observation group underwent intrathoracic internal fixation of rib fracture ends under total thoracoscopy. The magnified view provided by thoracoscopy allows for a clear assessment of the severity of the fracture, which is beneficial for removing accumulated blood and blood clots, thereby shortening the time required for thoracic exploration. The magnification effect of thoracoscopy also helps surgeons avoid intercostal vessels and nerves, reducing collateral damage from the surgery and minimizing intraoperative bleeding. Compared with percutaneous incisions, the incisions used in total thoracoscopic rib fixation surgery are smaller. During the fixation process, only the parietal pleura needs to be opened, avoiding extensive incisions that damage muscles, especially the thicker muscle tissues in the back. As a result, patients experience less postoperative pain, which facilitates recovery and reduces hospital stay duration. Additionally, traditional surgical incisions may face difficulties in fixing rib fractures that are too close to the scapula due to the obstruction by the scapula. Total thoracoscopic internal fixation can compensate for this shortcoming.

In this study, the total thoracoscopic group had shorter operative time, less intraoperative blood loss, and shorter postoperative hospital stay duration compared with the traditional incision group. Moreover, the pain index in the thoracoscopic group was lower than that in the traditional incision group at postoperative day 3 and day 5, and the partial pressure of oxygen was higher in the thoracoscopic group than in the traditional incision group at postoperative day 3 and day 5. There were no significant differences between the two groups in fracture healing status and incidence of complications. This indicates that total thoracoscopic MAEF internal fixation can effectively reduce surgical trauma, alleviate patient pain, improve pulmonary function, and facilitate postoperative recovery, achieving therapeutic effects comparable to those of open thoracotomy surgery. Additionally, the smaller incisions used in thoracoscopic surgery can also meet patients’ aesthetic needs.

After summarizing our findings, we identified that the advantages of total thoracoscopic MAEF internal fixation treatment are mainly reflected in the following aspects: [1] Intrathoracic fixation can avoid the difficulties in choosing surgical incisions for multiple rib fractures in traditional surgery, and the reduction of surgical incisions can also decrease postoperative pain for patients; [2] The MAEF is located on the internal surface of the rib, and after surgery, patients cannot palpate the internal fixation device through the chest wall, which reduces the foreign body sensation caused by subjective factors; [3] Traditional open surgery requires dissection of muscles such as the pectoralis major, pectoralis minor, and serratus anterior to expose the rib fracture ends, but total thoracoscopic surgery only requires dissection of the parietal pleura at the fracture site, avoiding the impact of cutting muscle attachment points on the patient’s chest wall and upper limb movement functions. Meanwhile, since the tissue at the parietal pleura is relatively thin, dissection is simpler, and there is less intraoperative bleeding, which can reduce surgical trauma; [4] Both total thoracoscopic MAEF internal fixation treatment and thoracic exploration are completed within the thoracic cavity, and thoracoscopic techniques are already well-established for thoracic surgeons, allowing them to quickly adapt to this surgical approach.

Although the thoracoscopic MAEF internal fixation treatment for rib fractures has advantages over traditional incisions, there are still some limitations: [1] This surgical approach requires incision of the parietal pleura, which may lead to pleural adhesions later on. This could potentially affect any future thoracic surgeries. [2] The existing internal fixation devices come in relatively fixed sizes, which may pose difficulties when applied to patients with unusual rib shapes or curvatures. [3] Special surgical instruments are required for this technique, and it takes some time for surgeons to become proficient with them. [4] Currently, the cost of this internal fixation device is higher than that of conventional internal fixation devices, placing a heavier economic burden on patients.

## Conclusion

The total thoracoscopic MAEF internal fixation surgery for rib fractures is easy to learn, causes minimal trauma, allows for rapid postoperative recovery, and achieves fracture healing comparable to that of open thoracotomy surgery.

## Data Availability

For further details, the corresponding author can be contacted.

## Abbreviations

MAEF: Memory alloy embracing fixator
CT: Computerized tomography
VAS: Visual analogue scale
AIS: Abbreviated injury scale
NSAIDs: Non-Steroidal anti-inflammatory drugs
PCA: Patient-controlled analgesia
ISS: Injury severity score
